# Cutaneous adverse effects associated with LAG‐3 inhibitor use in cancer treatment: A systematic review

**DOI:** 10.1002/ski2.296

**Published:** 2023-10-13

**Authors:** Hira Ghani, Samavia Khan, Marielle Jamgochian, Beth Richards, Erica DeCecco, Rebecca Fliorent, Nithisha Cheendalla, Khalil Khatri, Babar Rao

**Affiliations:** ^1^ Nassau University Medical Center East Meadow New York USA; ^2^ Center for Dermatology Rutgers Robert Wood Johnson Medical School Somerset New Jersey USA; ^3^ Cooper Medical School of Rowan University Camden New Jersey USA; ^4^ Rowan‐Virtua School of Osteopathic Medicine Stratford New Jersey USA; ^5^ Skin and Laser Surgery Center of New England Nashua New Hampshire USA; ^6^ Department of Dermatology Weill Cornell Medicine New York New York USA

## Abstract

Immunotherapy has become a mainstay of treatment for many cancers. Multiple immune checkpoint inhibitors have been used to treat malignancies, including anti‐programed death‐1 (PD1) and anti‐cytotoxic T‐lymphocyte‐associated protein (anti‐CTLA4). However, a significant percentage of patients develop resistance to these immunotherapy drugs. Therefore, novel strategies were developed to target other aspects of the immune response. Lymphocyte activation gene‐3 (LAG‐3) is a cell‐surface molecule found on natural killer cells and activated T‐cells which negatively regulates T‐cell proliferation and function. LAG‐3 inhibitors interact with LAG‐3 ligands on the surface of T‐cells to block T‐regulatory (Treg) cell activity, suppress cytokine secretion and restore dysfunctional effector T‐cells which subsequently attack and destroy cancer cells. This review reports the dermatologic side effects associated with LAG‐3 inhibitors used in the treatment of melanomas. Using PRISMA 2022 guidelines, a comprehensive literature review of PubMed, Google Scholar, Embase, Cochrane, and Web of Science databases was conducted. Three studies were identified that demonstrated that the use of LAG‐3 inhibitors, whether as a single agent or in combination with other immune checkpoint inhibitors, resulted in stomatitis, pruritus, rash, dry skin, erythema, and vitiligo. Further research is warranted to assess the cutaneous adverse events observed with LAG‐3 inhibitors in treating melanoma and to identify populations most vulnerable to such side effects.

## Graphical abstract

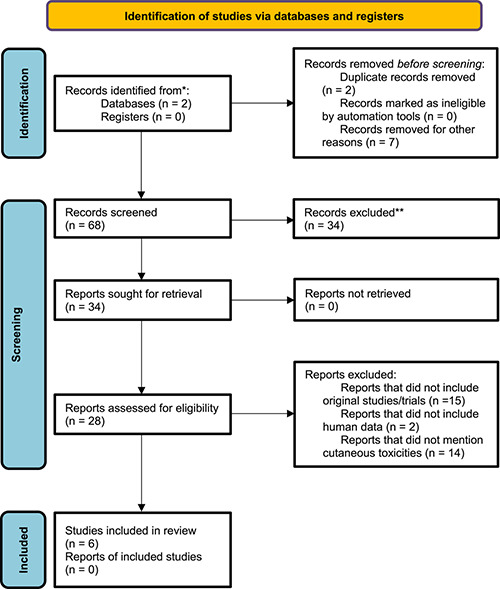

**What is already known about this topic**

- LAG‐3 inhibitors have been studied, as single agents or in combination with other checkpoint inhibitors, in the treatment of different skin cancers.

**What does this study add?**

- This study demonstrates that LAG‐3 inhibitors, whether as a single agent or in combination with other checkpoint inhibitors, may result in a variety of transient cutaneous conditions, including stomatitis, pruritus, dermatitis, dry skin, erythema, and vitiligo in the treatment of different cancers.

## 1 INTRODUCTION

Most patients with early‐stage melanoma are fully treated with resection of the primary lesion. Complications, such as metastasis and difficult‐to‐resect lesions, can make treatment plans more challenging.^1^, ^2^ Immunotherapy has recently gained increasing popularity in such complex circumstances, and it works by activating the host's immune system to suppress cancer cells. Checkpoint proteins, which are cell‐surface proteins that determine the magnitude and duration of immune responses, are common targets. Immunotherapy with checkpoint inhibitors, such as programed cell death protein‐1 (PD‐1) and anti‐cytotoxic T‐lymphocyte‐associated protein (CTLA‐4), has become a mainstay in the treatment of various cancers, including melanoma.^3^, ^4^, ^5^

Lymphocyte activation gene‐3 (LAG‐3), mainly found on natural killer cells and activated T‐cells, has recently been studied as a potential target for immunotherapy. LAG‐3 aims to inhibit T‐cell proliferation and function while promoting T‐regulatory cell (Treg) activity.^6^, ^7^ It is frequently overexpressed in cancer cells, allowing them to evade the immune system. A high number of LAG‐3 receptors on T‐cells has been associated with poor prognosis and decreased survival in cancer patients.^6^, ^7^ Recent clinical studies have looked at LAG‐3 inhibitors as a promising treatment for cancers, including melanoma. LAG‐3 inhibitors aim to promote effector T‐cell activity to attack cancer cells. However, they also inhibit Treg activity, which can induce reactions that present similar to autoimmune conditions.^7^, ^8^ This leads to several side effects associated with the use of LAG‐3 inhibitors.

As LAG‐3 inhibitors gain popularity in the clinical world to treat melanoma, more adverse effects have been reported. A common category of side effects comprises dermatologic reactions with a variety of presentations. In this systematic review, we analyse and summarise the most up‐to‐date literature to outline the cutaneous adverse events associated with LAG‐3 inhibitors.

## 2 METHODS

A comprehensive literature search was conducted using PubMed, Google Scholar, Embase, Cochrane, and Web of Science databases to select relevant articles using PRISMA (Preferred Reporting Items for Systematic Reviews and Meta‐Analyses) guidelines (2022). The initial search was conducted in May 2023, after which the titles and abstracts were screened for inclusion criteria by two independent reviewers (E.R., E.D.). Three reviewers (E.R., E.D., M.J.) reviewed full texts of shortlisted articles to ensure they met the inclusion criteria. Any disagreements were resolved by a fourth reviewer (H.G.). Following that, pertinent variables such as type of study, signs/type of cutaneous toxicity, patient demographics, a short description of the study, and effectiveness of treatment were extracted from each study.

### 2.1 Search criteria

Combinations of search terms were run in both databases. We used the following search string to identify relevant articles:

“lag3” AND “toxicity”: 58 results

AND

“lag3 inhibitor” AND “melanoma” AND “toxicity”: 9 results

AND

“relatlimab” AND “toxicity”: 1 result

Our search was limited to peer‐reviewed journal articles published in the last decade (2015–2023). We assessed article quality, study context and design, and outcomes. Inclusion criteria included original human studies that were on PubMed and Google Scholar, written in English language and those that specifically mentioned the dermatological toxicities seen with LAG‐3 inhibitors.

Exclusion criteria involved studies that were not accessible for full‐text review, those without a discussion of cutaneous toxicities, and those which were not original clinical trials involving humans.

A total of 68 records were generated using the above search terms, out of which 34 studies were extracted. After screening the titles/abstracts and full texts for inclusion criteria, a final number of 6 studies were shortlisted for our systematic review.

## 3 RESULTS

**FIGURE 1:**
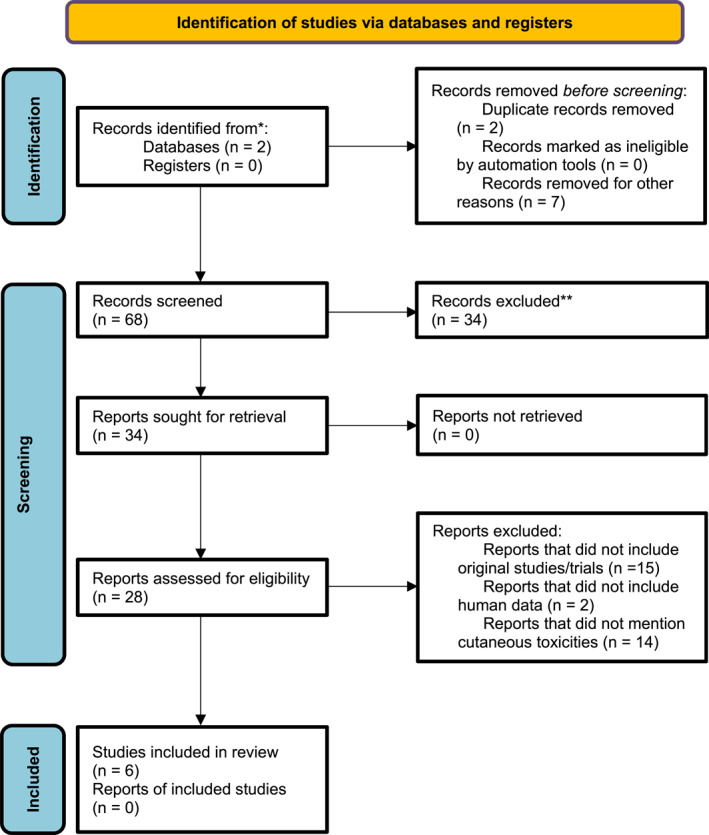
Study identification, screening, and inclusion criteria. *From:* Page MJ, McKenzie JE, Bossuyt PM, Boutron I, Hoffmann TC, Mulrow CD, et al. The PRISMA 2020 statement: an updated guideline for reporting systematic reviews. BMJ 2021; 372:n71. doi: 10.1136/bmj.n71.

Study selections are detailed in Figure 1. Three studies resulted after eliminating duplicates and following exclusion criteria.

Study selections are detailed in Figure 1. Four studies resulted after eliminating duplicates and following exclusion criteria.

Of the 6 studies identified in the systematic review, all were clinical trial data from phase I, Phase I/II or Phase II/III trials. The median ages of subjects in the three included studies were 63 years, 63.5 years, and 58 years, respectively. The age range of patients included in the study was 19–94 years. A total of 621 patients taking LAG‐3 were identified. Among the 621 subjects, the most common adverse effects noted were pruritus and rash. A total of 102 (16.4%) subjects experienced pruritus and approximately 114 (18.3%) experienced rash, “immune mediated rash” or maculopapular rash. In Tawbi et al trial, 37 (10.4%) participants experienced vitiligo.^5^ Other cutaneous adverse effects noted in the Schoffski et al trial included stomatitis (*n* = 6, 2.4%), dry skin (*n* = 4, 1.6%), and erythema (*n* = 4, 1.6%), but these were seen in a minority of trial subjects.^9^ The design of the studies was heterogeneous as each had different endpoints. Wang‐Gillam et al study primarily looked for early safety data and identified rash as the only cutaneous adverse effect in this small group.^10^ Schoffski et al, Tawbi et al, Sibaud et al, and Belum VR et al studies were able to further identify cutaneous adverse effects of these medications, including vitiligo, stomatitis, dry skin and erythema, and pruritus.^5^, ^9^, ^11^, ^12^ Because the study groups included patients with a variety of different diseases, it is hard to specifically generalise these results to patient populations with melanoma who may be treated with this newly emerging drug class. An analysis of the types of studies and their summative reported adverse effects are highlighted in Table 1.

**TABLE 1:** Summative analysis of studies and reported cutaneous adverse effects.

Author, year	Study type	Number of patients	Disease	Demographics	Therapy	Cutaneous adverse effects
Tawbi et al 2022	Phase II/III RCT	355	Melanoma	‐Median age 63 ‐Range 20–94 210 (59.2%) M 145 (40.8%) F	Relatlimab and Nivolumab (*N* = 355) vs Nivolumab alone (*N* = 359)	Pruritus (*N* = 83) (23.4%) rash (*N* = 55) (15.5%) vitiligo (*N* = 37) (10.4%) “immune‐mediated” rash (*N* = 33) (9.3%)
Wang‐Gillam et al 2013	Phase I study	17 (11 taking LAG3)	Advanced pancreatic adenocarcinoma being treated with gemcitabine	‐Median age 63.5 ‐Range 36–90 9 (53%) M, 8 (47%) F	Gemcitabine + IMP321 at 0.5 mg *N* = 6, gemcitabine + IMP321^a^ at 2 mg *N* = 5 *N* = 6 treated with gemcitabine alone	Of 5 patients who received 2 mg dose of IMP321^a^, 1 experienced rash. No patients in the gemcitabine + IMP321^a^ 0.5 mg group had reported adverse reactions.
Schoffski et al 2022	Phase I/II RCT	255	Advanced/metastatic solid tumours progressing or unsuitable for standard‐of‐care therapy (most commonly NSCLC (*N* = 28), colorectal cancer (*N* = 21), and cutaneous melanoma (*N* = 18))	Single agent: ‐Median age 59 ‐Range 26–81 65 (48.5%) M 69 (51.5%) F Combination: Median age 58 Range 19–77 55 (45.5%) M 66 (54.5%) F All subjects: ‐Median age 58 ‐Range 19–81 120 (47.1%) M 135 (52.9%) F	Single agent: Ieramilimab (*N* = 134) vs Combination: Ieramilimab + spartalizumab (*N* = 121)	Single agent: Pruritus (*N* = 7) (5.2), Rash (*N* = 4) (3%), maculopapular rash (*N* = 2) (1.5%), stomatitis (*N* = 1) (0.7%), dry skin (*N* = 3) (2.2%), erythema (*N* = 1) 0.7% Combination: Pruritus (*N* = 12) (9.9%), rash (*N* = 10) 8.3%, maculopapular rash (*N* = 9) 7.4%, stomatitis (*N* = 5) 4.1%, dry skin (*N* = 1) 0.8%, erythema (*N* = 3) 2.5% All subjects: Pruritus (*N* = 19), (7.5%), rash (*N* = 14), (5.5%), maculopapular rash (*N* = 11) (4.3%), stomatitis (*N* = 6) (2.4%), dry skin (*N* = 4) (1.6%), erythema (*N* = 4) (1.6%)
Sibaud 2017	Review of 6 Phase I–III studies		Advanced melanoma		Nivolumab, anti‐PD‐1	Rash (13%–21.5%) Pruritus (17%–19%) Vitiligo (7.5%–10.5%)
					Ipilimumab, anti‐CTLA‐4	Rash (14.5%–26%) Pruritus (24.5%–35.5%) Vitiligo (1.5%–8.5%)
					Nivolumab + ipilimumab	Rash (28.5%–55%) Pruritus (33%–47%) Vitiligo (6.5%–11%)
Belum VR et al 2016	Review of 8 8 Phase I–III studies for Nivolumab, 5 Phase 1 studies for Pembrolizumab	1136–Nivolumab			Nivolumab	All‐grade rash (14.3%) Pruritus (13.2%) Vitiligo (7.5%)
		177–Pembrolizumab			Pembrolizumab	All‐grade rash (16.7%) Pruritus (20.2%) Vitiligo (8.3%)
Hwang et al 2016	A single institutional cohort at Westmead hospital, Sydney, Australia	82	Metastatic melanoma		Anti‐PD1 agents	Vitiligo (15%) Lichenoid reactions (17.1%) Eczema (17.1%) Actinic Keratosis (13.4%) Pruritus (11%)

^a^ IMP321 is a recombinant soluble LAG‐3‐Immunoglobulin fusion protein.

## 4 DISCUSSION

LAG‐3 inhibitors used in combination with other immunotherapy drugs as in Tawbi et al and Schoffski et al trials, increased the incidence of cutaneous adverse events, regardless of whether it concurrent with a checkpoint inhibitor or solo‐therapy.^5^, ^9^ Per Schoffski et al, the only dermatological side effect that decreased with combination therapy compared to LAG‐3 inhibitor alone was the occurrence of dry skin.^9^ Findings of Wang et. al indicated one additional case of dermatitis upon increasing the dose of LAG‐3 inhibitor from 0.5 to 2 mg (mg); however, due to the limited sample size (*n* = 11), it is difficult to draw any conclusions.^10^

Of the six studies analysed, none focused on cutaneous adverse events as a primary endpoint. Therefore, it is possible other cutaneous side effects of LAG‐3 inhibitors remain unreported. Dermatological side effects are among the most prevalent adverse effects of anti‐cancer immunotherapy drugs. These effects have been extensively reported, including acneiform rash, alopecia, vitiligo, xerosis, pruritus, eczema‐like or psoriatic lesions, lichenoid dermatitis, nonspecific maculopapular rash, oral mucositis, paronychia, onycholysis, and even induction of secondary skin tumours such as squamous cell carcinoma and keratoacanthomas.^11^, ^12^, ^13^, ^14^, ^15^, ^16^, ^17^ Comparatively, adverse events of the skin encompassed within the evaluated studies were predominately limited to rash, pruritus, erythema, dry skin, and stomatitis, with the exception of Schoffski et al, Tawbi et al, Sibaud et al, Belum VR et al, and Hwang et al, who also discussed vitiligo.^5^, ^9^, ^11^, ^12^, ^16^ Belum VR et. al study found that all reports of vitiligo were associated with trials investigating melanoma.^12^ A previous study found immunotherapy‐induced vitiligo depigmentation in patients with melanoma correlated with a reduced risk of disease progression and increased survival.^5^, ^18^, ^19^ Thus, we emphasise the importance of documentation of dermatological adverse effects, as they may potentially be prognostic of treatment outcomes, and inform treatment management. To summarise, dermatological effects secondary to LAG‐3 inhibitor use are apparent in patients with cancer. Additional research is required with regard to the demographics of patients who experience cutaneous adverse effects when specifically treated with LAG‐3 inhibitors for melanoma.

## 5 CONCLUSION

Findings regarding cutaneous adverse effects of LAG‐3 inhibitors are limited. Per our systematic review, we identified six studies that demonstrated that the use of LAG‐3 inhibitors, whether as a single agent or in combination with other immune checkpoint inhibitors, resulted in a variety of dermatological conditions, including stomatitis, pruritus, rash, dry skin, erythema, and vitiligo. Further research is warranted to assess the cutaneous adverse events observed with the use of LAG‐3 inhibitors in treating melanoma and to identify populations most vulnerable to such side effects.

## CONFLICT OF INTEREST STATEMENT

None to declare.

## AUTHOR CONTRIBUTIONS

**Hira Ghani**: Writing – original draft (equal); Writing – review & editing (equal). **Samavia Khan**: Writing – original draft (equal); Writing – review & editing (equal). **Marielle Jamgochian**: Writing – original draft (equal); Writing – review & editing (equal). **Beth Richards**: Supervision (equal). **Erica DeCecco**: Writing – original draft (equal); Writing – review & editing (equal). **Rebecca Fliorent**: Writing – original draft (equal); Writing – review & editing (equal). **Nithisha Cheedalla**: Writing – original draft (equal); Writing – review & editing (equal). **Khalil A Khatri**: Writing – original draft (equal); Writing – review & editing (equal). **Barbar Rao**: Writing – original draft (equal); Writing – review & editing (equal).

## ETHICS STATEMENT

Not applicable.

## Data Availability

Data sharing not applicable to this article as no datasets were generated or analysed during the current study.
